# Pollutant particle priming amplifies airway compartment specific neutrophil and macrophage inflammatory programs following house dust mite acute exposure

**DOI:** 10.3389/fimmu.2026.1869644

**Published:** 2026-08-17

**Authors:** Kirsty Meldrum, Martin Oliver Leonard

**Affiliations:** Toxicology Department, UK Health Security Agency, Harwell Campus, Chilton, United Kingdom

**Keywords:** allergen, inflammation, luminal airway, lung, particle, pollutant, priming, single cell sequencing (scRNA-seq)

## Abstract

Real world inhalation exposures occur as temporally staggered mixtures of environmental pollutants and indoor allergens, yet the mechanistic consequences of sequential exposure remain poorly defined. Here, we investigated whether diesel exhaust particle (DEP) exposure primes the lung to alter subsequent responses to house dust mite (HDM) allergen independently of direct co-exposure or preexisting sensitisation. Using a murine model with defined temporal separation between exposures, we combined compartment resolved bulk transcriptomics and targeted single cell profiling to map inflammatory and cellular responses across lung tissue and airway lumen. DEP priming significantly amplified leukocyte recruitment following HDM challenge, with a dominant increase in neutrophils and no corresponding eosinophilic response. Transcriptomic analyses revealed that DEP alone preferentially induced NF-κB associated inflammatory programs, while HDM triggered both NF-κB and interferon (IFN) associated transcriptional responses. Importantly, DEP priming qualitatively reprogrammed the allergen response, enhancing both IFN stimulated gene (ISG) expression and pro-inflammatory cytokine networks. These effects were most pronounced in the airway luminal compartment, indicating a prominent inflammatory niche shaped by recruited immune cells. Single cell analyses identified expansion and activation of multiple neutrophil subpopulations, alongside recruitment of macrophage subsets, NK cells, and CD8^+^ T cells. Ligand receptor inference and protein measurements implicated Cxcl1/Cxcl2–Cxcr2 signalling as a central axis driving neutrophil recruitment, with additional Ccr5 and Cxcr3 linked pathways contributing to broader immune cell infiltration. *Ex vivo* stimulation suggested that DEP priming enhances intrinsic cellular responsiveness to HDM, in addition to increasing the pool of responsive cells. Collectively, these findings demonstrate that pollutant exposure reprograms airway immune landscapes to amplify subsequent allergen responses, suggested through neutrophil centric and interferon linked mechanisms. This work provides a mechanistic framework for sequential exposure risk, highlights the importance of compartmentalised immune dynamics, and informs the design of advanced *in vitro* models for respiratory hazard assessment.

## Introduction

A central challenge in respiratory inhalation research is that real-world exposures are heterogeneous, mixed, and temporally staggered rather than occurring as isolated events, as individuals move repeatedly between traffic-related outdoor microenvironments and indoor spaces (1, 2). Importantly, although the greatest cumulative exposure to traffic-derived air pollutants in urban residences may occur at home, peak intensity is greatest during commuting and transit through traffic-heavy microenvironments (1, 3, 4). In contrast, indoor settings present a different profile of inhalation hazards, with aeroallergens such as house dust mite (HDM) highly prevalent, and homes containing higher levels than outdoor and public spaces (2, 5, 6). Indeed, HDM is the most common aeroallergen to which humans are sensitised (7), with exposure linked to asthma exacerbation especially in children (8). Pollutant exposure is also a major trigger for asthma exacerbations, with components such as PM2.5 identified as causative (9). While studies have investigated interactions between particulate pollutants in the development and exacerbation of allergic asthma using simultaneous hazard exposure models (10–12), further work is needed to fully understand how sequential exposure scenarios impact disease risk. This is especially important because the highest air pollution and indoor allergen exposures often occur sequentially, separated by hours to days.

Experimental studies have investigated several sequential exposure scenarios. For example, a recent study found that maternal diesel exhaust particle exposure increased offspring susceptibility to allergic airway disease by inducing a natural killer (NK) cell dependent, early life proallergic response (13). Similarly, early life exposure to combustion particulates, although initially immunosuppressive, resulted in severe allergic inflammation in adults, attributable to changes in the adaptive immune system (14). These and similar longer interval studies (15) suggest that pollutant exposure can induce changes in innate and adaptive immunity that confer longer-term cellular memory and alter subsequent allergen handling. Studies examining pollutant priming before subsequent allergen challenge have also been carried out using human exposure chamber approaches. For example, diesel exhaust particles (DEP), when administered to atopic individuals before allergen exposure in a repeat exposure protocol, can act as a mucosal adjuvant, augmenting subsequent allergen specific IgE production (16). In another set of studies, atopic individuals were exposed to diesel exhaust 1 hr before allergen administration (17–19). Allergic endpoints (eosinophils, IL-5, ECP) as well as non-allergic endpoints (neutrophils, IL-8, MCP-1) were increased following subsequent allergen exposure (17). This was accompanied by increases in the neutrophil associated effector protein MPO (18) as well as enhanced airway epithelial allergen induced inflammatory proteins (CST2) and airway mucins (19). While important in demonstrating that model pollutant material enhances allergen induced inflammatory responses, these latter studies may be considered co-exposures and may not involve a sufficient interval to exclude acellular particle–allergen physical interaction as a mechanism for enhanced effects.

Studies of sequential administration of DEP and allergen have also been examined in models pre-sensitised to allergen (20, 21). However, unlike these studies, the extent to which acute changes in inflammatory cell composition (population dynamics within the lung) and resident cell responsiveness, independent of a pre-existing or developing allergen sensitisation microenvironment, prime tissues for subsequent allergen responsiveness remains poorly defined, particularly over the hours to days window in which the primary exposure is likely to reshape cellular composition and responsiveness. This is important because in an acute unsensitised setting, pollutant enhanced responses to HDM may occur before *de novo* allergen specific IgE/IgG responses develop and may instead be initiated by innate cues within complex HDM exposures, including protease active allergens, endotoxin/TLR4 ligands and HDM associated double stranded RNA that activates TLR3 dependent interferon signalling (22–24).

Contemporary mucosal immunology increasingly recognizes airway epithelial cells as active immunological sentinels that integrate environmental cues and coordinate barrier repair, chemokine release, and leukocyte recruitment (25–27).. In parallel, resident and recruited myeloid populations, particularly alveolar macrophages and monocyte-derived macrophages shape the threshold for inflammation and the quality of downstream adaptive responses (28). Alveolar macrophage sin particular are strategically positioned to clear inhaled particles, including allergens and to communicate signals to surrounding cells to direct subsequent inflammatory responses (29). Similarly, neutrophil (NEU) recruitment during the early phase of inflammatory tissue responses in the lung plays a central role in shaping subsequent responses to environmental cues (30, 31). Importantly, even when an allergen response is classified as type 2 dominated, airway inflammation is heterogeneous. A clinically significant subset of asthma is characterized by airway neutrophilia, reduced corticosteroid responsiveness, and association with infections and environmental toxicants (32, 33), highlighting the relevance of neutrophilic mechanisms of disease. Indeed, neutrophils have been suggested to mediate DEP exacerbation potential in a model of allergen co-exposure (34) as well as to contribute to enhanced allergic sensitisation and TH17 type inflammation (35). Recruitment of neutrophils to the lung by DEP was identified as mediated through the Cxcl1/Cxcl2 – Cxcr2 chemokine axis and blockade of these chemokine signals reduces DEP enhanced allergic inflammation (30, 31).

Key gaps remain however, that directly limit hazard characterisation in these complex multicellular tissues and exposure scenarios. Many studies emphasize whole lung or single compartment readouts, yet airway luminal immune niches and parenchymal tissue can exhibit distinct cell compositions and signalling constraints, particularly in the context of neutrophil trafficking and interferon/NF-κB pathway activation (25, 36). Identifying which cell types carry pollutant-conditioned transcriptional programmes in priming scenarios, as well as changes in the types of cells recruited by different exposures, is essential for translating animal exposure biology into human-relevant *in vitro* systems. Advanced airway models are increasingly positioned to test different components of air pollutant material for relative hazard, but their predictive value depends on incorporating the relevant multicellular interactions, including myeloid granulocyte epithelial circuits (37, 38). Finally, cumulative and sequential exposure framing, central to real world risk requires mechanistic endpoints that can map appropriate hazard scenarios, where one exposure meaningfully modifies the response to the next.

To address these knowledge gaps, we applied a sequential exposure paradigm of an initial DEP exposure with an interval of 24 hours prior to HDM administration allowing clear definition of priming mechanisms independent of potential direct pollutant-allergen interaction and independent of pre-existing sensitisation inflammatory conditions, that models early events in pollutant enhanced allergen responsiveness. Through integrating airway compartment resolved bulk RNA sequencing with targeted single-cell profiling we examined how pollutant particle priming modifies acute allergen responses across the lung mucosa and airway lumen. By identifying the dominant pathways and cell to cell communication modules induced by sequential exposures, our work aims to clarify mechanistic links between pollutant exposure and neutrophil centric airway inflammation, provide a systems level basis for complex hazard mitigation strategies, and inform the rational design of human *in vitro* models capable of reproducing pollutant allergen interactions relevant to respiratory immunotoxicology.

## Methods

### Animal procedures & bronchoalveolar lavage cell counts

DEP were sourced from the US National Institute of Standards and Testing (Gaithersburg, Maryland, USA) (Cat# SRM2975). Particles were prepared in PBS with or without HDM (Greer Laboratories, Lenoir, cat# XPB82D3A25, Lot# 327874). Particle preparations were dispersed by sonication (QSonica Sonicators, CT, USA) with 4.2x10^5^ kJ/m^3^ immediately prior to lung exposure. Female BALB/c mice between 6-8 weeks (Envigo, UK) were anaesthetised with 5% isoflurane/95% oxygen using a precision vaporizer. Under anaesthesia mice were administered with 25 µl/animal suspensions of either DEP (2mg/mL), HDM (1mg/mL) in droplets directly onto the nose. A 25 µl volume of PBS was used for control (CTL) exposures. Animals were then euthanised using 0.1mL sodium pentobarbital (200 µg/mL) by intraperitoneal injection and exsanguination by cardiac puncture. Bronchoalveolar lavage (BAL) was performed after tracheal catheterisation using three washes of 500 µl ice cold sterile PBS. Luminal airway cells (LAC) were defined as the cells recovered from these three pooled BAL washes, whereas lung tissue (TISS) comprised the post lavage lung. Lavage material was centrifuged at 1500 x g for 10 mins at 4 °C and the supernatant from the first lavage was collected for subsequent protein analysis by ELISA. BAL cells from all three lavage collections were combined in 1 ml PBS and counted using a haemocytometer for total cell counts. 700ul of this suspension was further centrifuged at 4 °C and lysed with RLT buffer for RNA-Seq analysis. The remaining cells were centrifuged in triplicate using EZ single cytofunnels (ThermoFisher Scientific, UK, cat# 97200125) onto Superfrost™ Plus slides (Menzeil-Gläser Superfrost Plus, ThermoFisher Scientific, UK, cat# 10149870) using a cytospin centrifuge. Differential immune cell counting was carried out using a Wright-Giemsa staining (Kwik Diff, ThermoFisher Scientific, UK, cat# 9990700) protocol before mounting and quantification of macrophage (MAC), lymphocyte (LYM), neutrophil (NEU) and eosinophil (EOS) cell numbers. Cells were counted for each animal over three different slides (~300 cells/slide). Lung tissue was subsequently removed, washed in sterile PBS and snap frozen for later analysis. All procedures comply with ARRIVE 2.0 essential guidelines and were performed in accordance with the Animals (Scientific Procedures) Act 1986 (under Project licence number P21FEA4BB), which complies with EU Directive 2010/63/EU. All experimental procedures were reviewed and approved by the local Animal Welfare and Ethical Review Body.

### Exposure protocols

**Figure 1:**
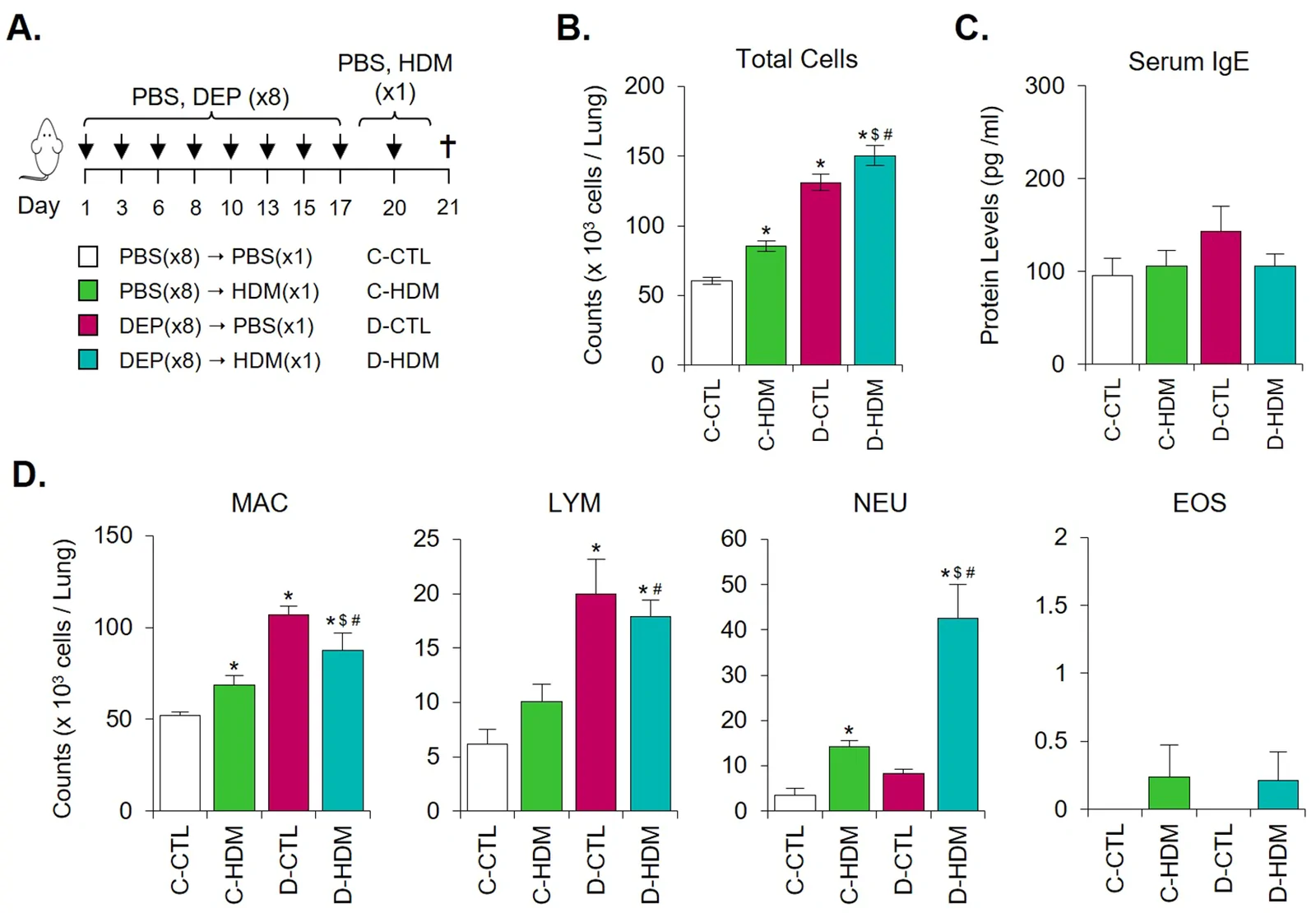
Effect of pollutant particle priming on HDM induced inflammatory responses within the lung. Mice (n=6-7 per group) were administered intranasally with either saline (PBS) or DEP (2.5 mg/kg) eight times interspersed over 17 days, followed by a single administration of PBS or HDM (1.25 mg protein/kg) for 24hrs, according to the combinations displayed **(A)**. BAL cells were collected from the lung and examined for total **(B)** or differential cell populations (MAC; Macrophages, LYM; Lymphocytes, NEU; Neutrophils, EOS; Eosinophils) **(D)** through microscopic analysis. Total plasma levels for IgE protein were also examined, using ELISA **(C)**. Results are expressed as Mean +/- SEM for each treatment group. Statistical comparisons between treatments were carried out by ANOVA with Fischer LSD test with significance indicated as * (p<0.05) when each treatment is compared to C-CTL; $ is D-HDM v D-CTL. # is D-HDM v C-HDM.

**Figure 2:**
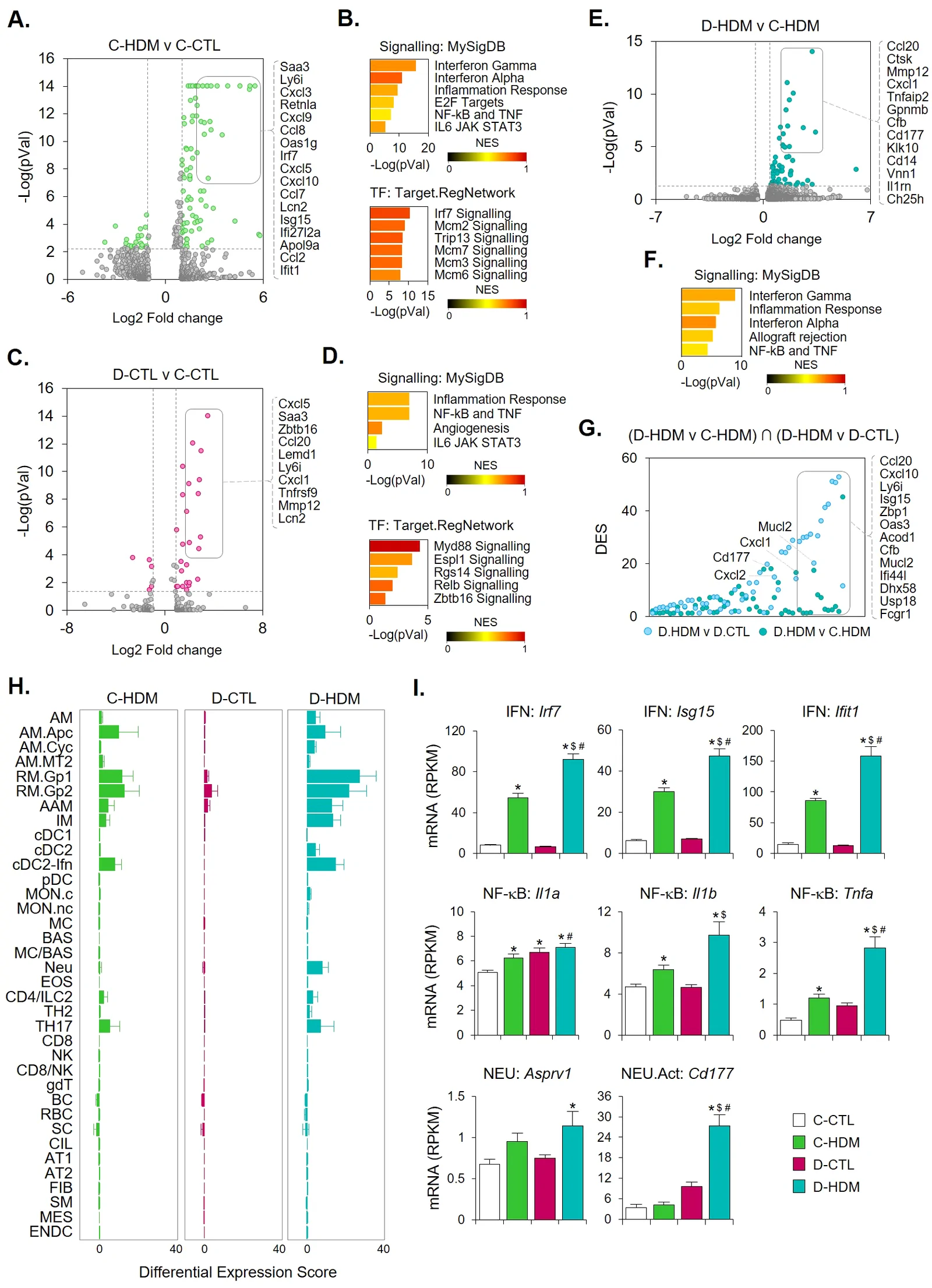
Transcriptional profiling of DEP priming effects on HDM induced changes in the lung tissue compartment. Mice (N = 4-5) from the same experiment as described in **Figure 1A** were processed for bulk-seq transcriptomic analysis of lung tissue. Differentially expressed genes (DEG) (> 2-fold change (FC); FDR p value < 0.05) were identified for C-HDM v C-CTL **(A)** and D-CTL v C-CTL **(C)** and displayed within volcano plots as green- and maroon-coloured dots, respectively. A list of the top upregulated genes in these comparisons is also displayed (A,C). Pathway analysis of DEG was also carried against cellular signalling and transcription factor regulated gene databases for C-HDM **(B)** and D-CTL **(D)** treatments. For comparisons of D-HDM v C-HDM treatments **(E)** a reduced fold change cut-off (> 1.25 up or down regulated) was used, with significant genes depicted as teal-coloured dots. Pathway analysis for differentially expressed genes (DEGs) in **(E)** are displayed in **(F)**. DEGs common to both D-HDM v C-HDM and D-HDM v D-CTL comparisons (>1.25 FC; p<0.05) were identified and displayed as differential expression score (DES) versus C-CTL levels for each original comparison derived list **(G)**. A list of the most upregulated genes from this comparison is also displayed **(G)**. Cell interpolation analysis of bulk-seq data was carried out against a database of cell type specific markers **(H)**. Results are expressed as mean +/- SEM of DES for genes within each cell type category. Cell type abbreviations are indicated in the abbreviations section. Select gene markers for pathway and cell type are also displayed (IFN; Interferon. NF-κB. NEU; Neutrophil. NEU.Act; Activated neutrophil) from RNA-Seq data as RPKM values **(I)**. Statistical comparisons for **(I)** used ANOVA with Fisher LSD test (* is <0.05 compared to C-CTL. $ is D-HDM v D-CTL. # is D-HDM v C-HDM).

**Figure 3:**
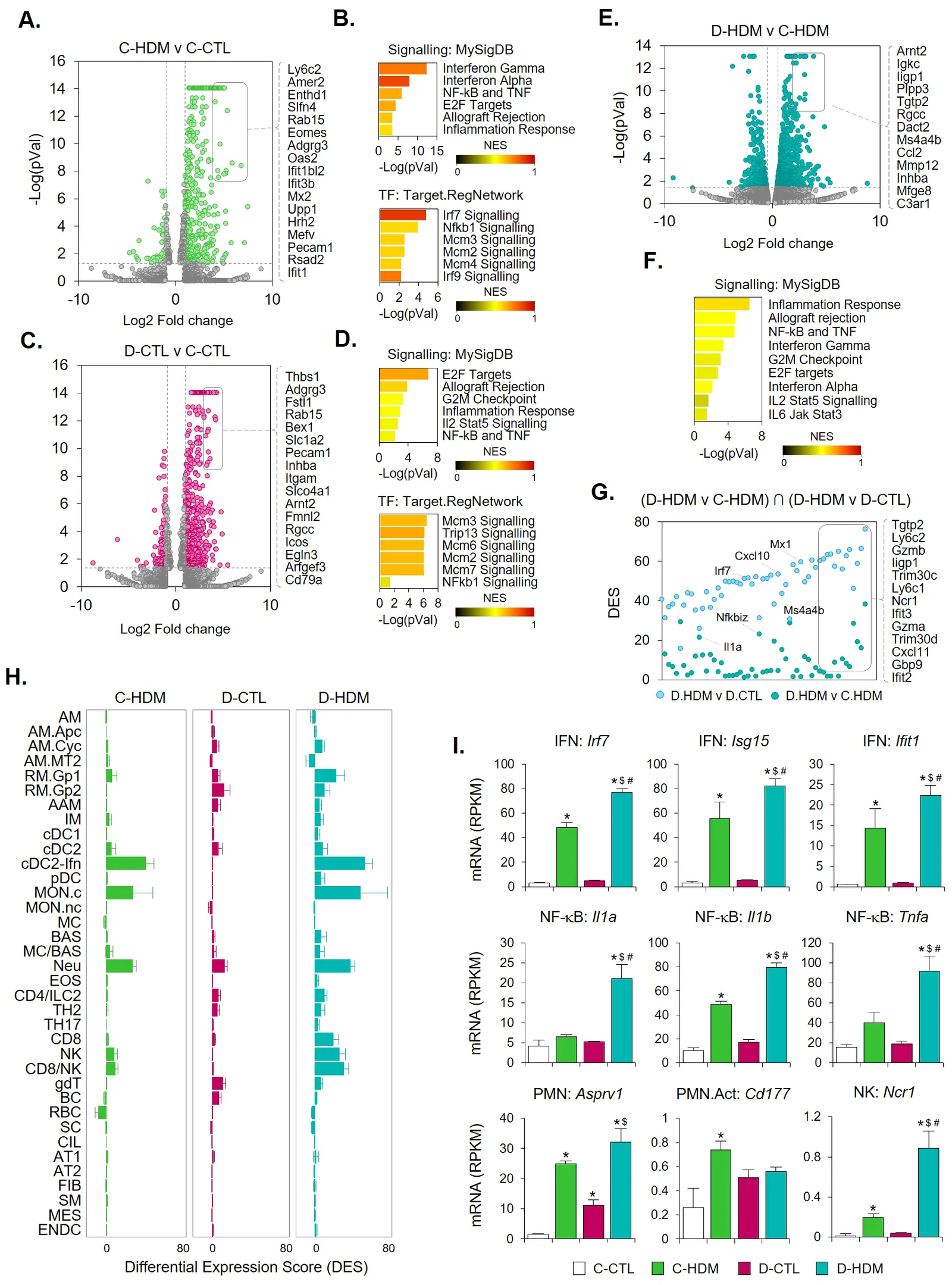
Transcriptional profiling of DEP priming effects on HDM induced changes in the lung luminal airway cell compartment. Mice (N = 3-5) from the same experiment as described in **Figure 1A** were processed for bulk-seq transcriptomic analysis of luminal airway cells extracted using bronchoalveolar lavage. Differentially expressed genes (DEG) (> 2-fold change (FC); FDR p value < 0.05) were identified for C-HDM v C-CTL **(A)** and D-CTL v C-CTL **(C)** and displayed within volcano plots as green- and maroon-coloured dots, respectively. A list of the top upregulated genes in these comparisons is also displayed (A,C). Pathway analysis of DEG was also carried against cellular signalling and transcription factor regulated gene databases for C-HDM **(B)** and D-CTL **(D)** treatments. For comparisons of D-HDM v C-HDM treatments **(E)** a reduced fold change cut-off (> 1.25 up or down regulated) was used, with significant genes depicted as teal-coloured dots. Pathway analysis for DEGs in **(E)** are displayed in **(F)**. DEGs common to both D-HDM v C-HDM and D-HDM v D-CTL comparisons (>1.25 FC; p<0.05) were identified and displayed as differential expression score (DES) versus C-CTL levels for each original comparison derived list **(G)**. A list of the most upregulated genes from this comparison is also displayed **(G)**. Cell interpolation analysis of bulk-seq data was carried out against a database of cell type specific markers **(H)**. Results are expressed as mean +/- SEM of DES of genes within each cell type category. Cell type abbreviations are indicated in the abbreviations section. Select gene markers for pathway and cell type are also displayed (IFN; Interferon. NF-κB. NEU; Neutrophil. NEU.Act; Activated neutrophil. NK; NK cell) from RNA-Seq data as RPKM values **(I)**. Statistical comparisons for **(I)** used ANOVA with Fisher LSD test (* is <0.05 compared to C-CTL. $ is D-HDM v D-CTL. # is D-HDM v C-HDM).

**Figure 4:**
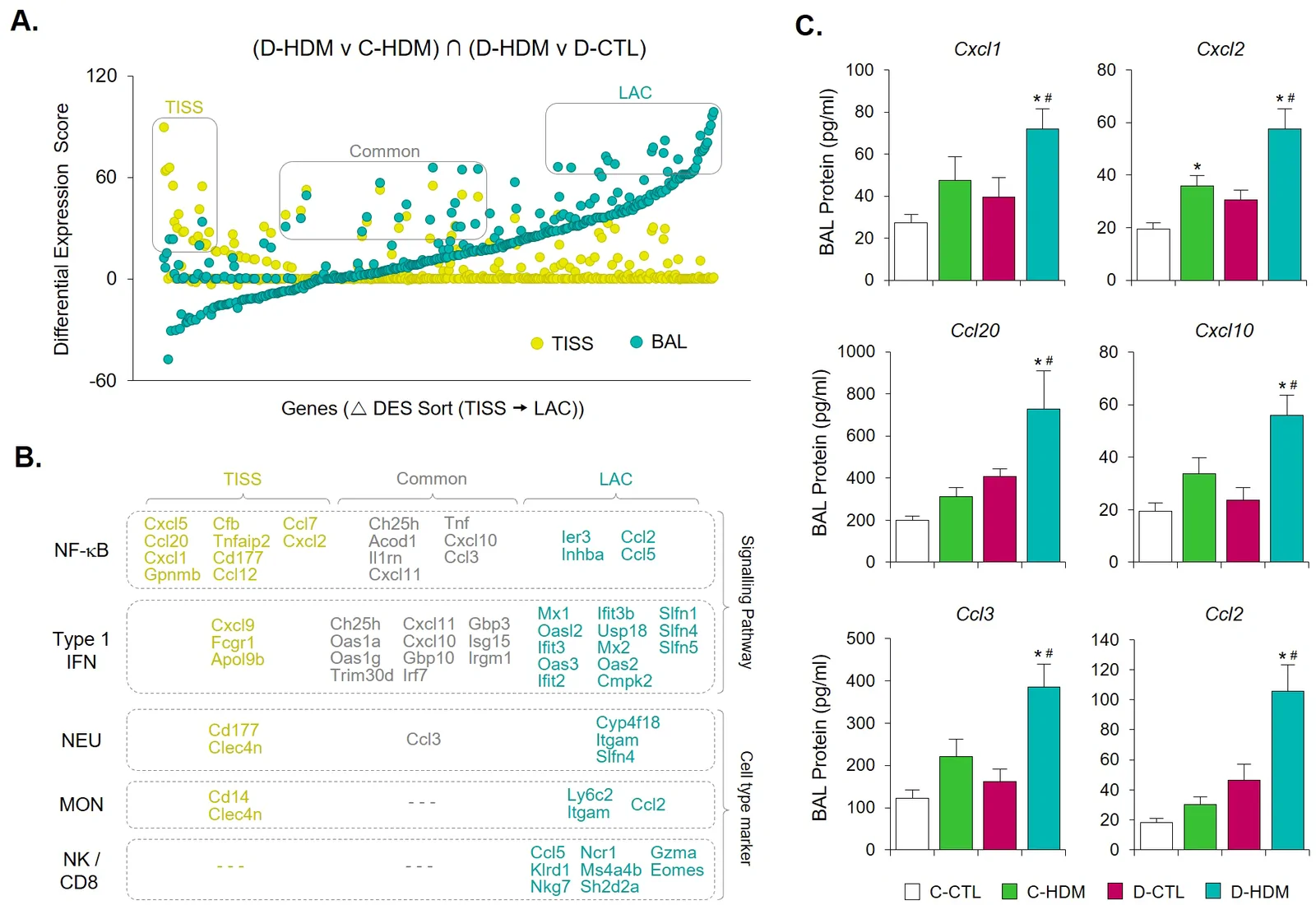
Enhanced DEP primed HDM transcriptional responses display airway lumen and tissue compartment dependent expression. Mice (N = 3-5) from the same experiment as described in **Figure 1A** were processed for bulk-seq transcriptomic analysis of lung LAC and TISS compartments. HDM enhanced genes after DEP priming were identified as having greater than 1.25-fold change (FC) with an FDR q value < 0.05, for both D-HDM v C-HDM and D-HDM v D-CTL treatment group comparisons. **(A)** Differential expression scores of D-HDM v C-CTL for these genes are displayed for both TISS and LAC datasets, sorted based on the difference between compartments. **(B)** The most significantly upregulated genes from panel A selective for LAC, TISS and common responsiveness were categorised for signalling pathway and cell type marker association. **(C)** Chemokine protein levels in BAL across treatment groups were analysed using ELISA. Statistical comparisons used ANOVA with Fisher LSD test (* = <0.05 compared to C-CTL. # = D-HDM v C-HDM).

**Figure 5:**
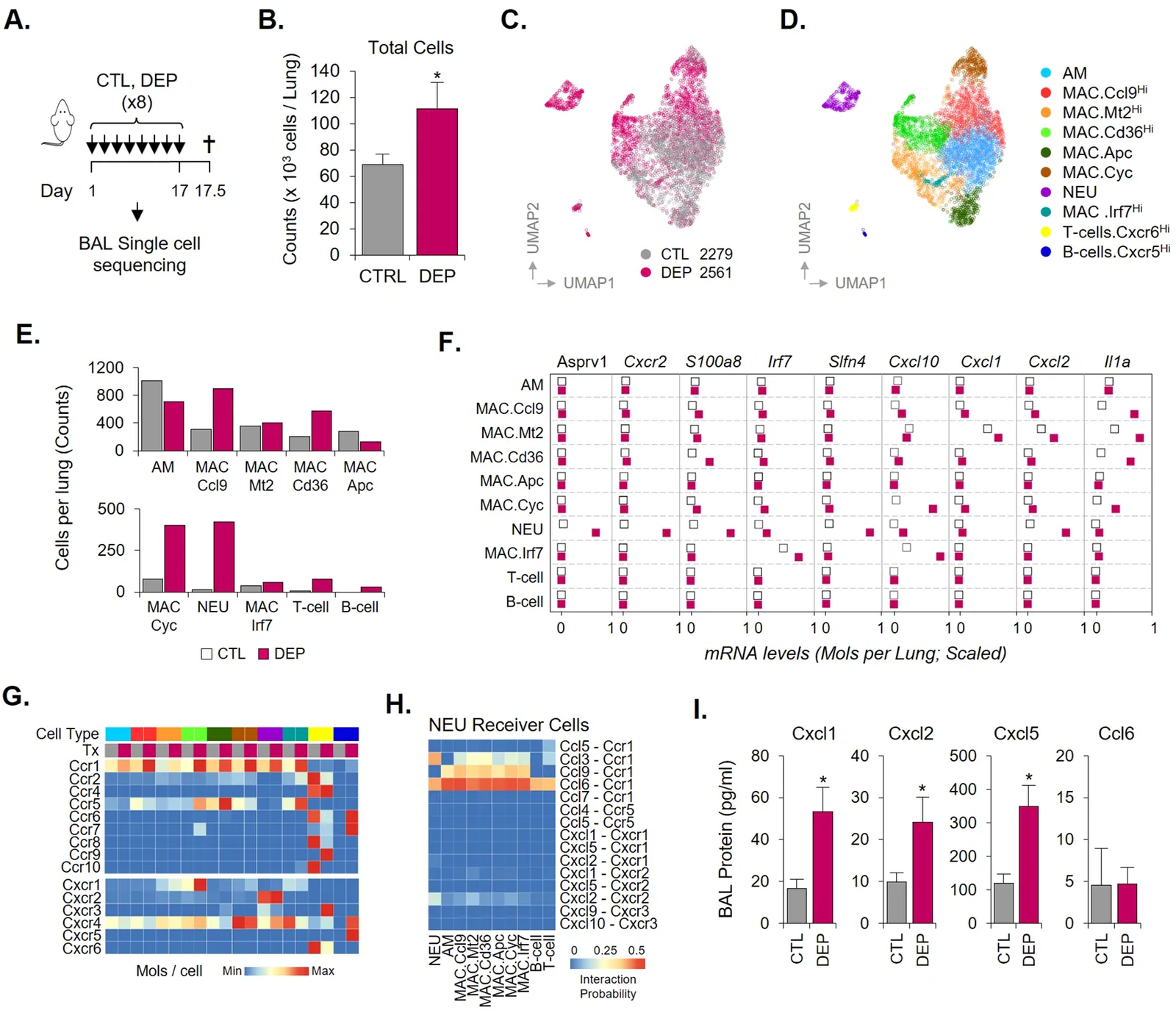
Repeat DEP exposure enhances LAC neutrophil recruitment and associated chemokine signals. Mice (n=3 per group) were instilled with 8 repeat exposures of DEP (2.5 mg/kg) over 17 days with collection of BAL cells 12 hrs after the last treatment **(A)**. Total cells within the lavage were examined by microscopy **(B)**. LAC CTRL and DEP cell populations (pooled n=3 per treatment) were examined for cell type marker and inflammatory gene expression using TGE ScSeq analysis **(C-H)**. ScSeq data was visualised as UMAP plots of cells per treatment group **(C)** or cell type **(D)**. Adjusted LAC cell counts normalised for total lung content are displayed **(E)** together with total LAC mRNA levels per lung for select genes **(F)**. Results are scaled to maximum levels for each gene for visual comparison across cell types **(F)**. Expression levels for chemokine receptor mRNA across cell types (Colour key as per **Figure 5D**) and treatment (Tx) are displayed as normalised expression to maximum for each gene **(G)**. CellChat analysis was used to identify ligand – receptor interaction pairs underpinning cell type specific communication, with strength of association indicated as interaction probability for NEU as the chemokine receptor expressing signal receiver cell population **(H)**. Chemokine protein levels in BAL across treatment groups were analysed using ELISA **(I)**. Statistical comparisons used ANOVA with Fisher LSD test (* = <0.05).

**Figure 6:**
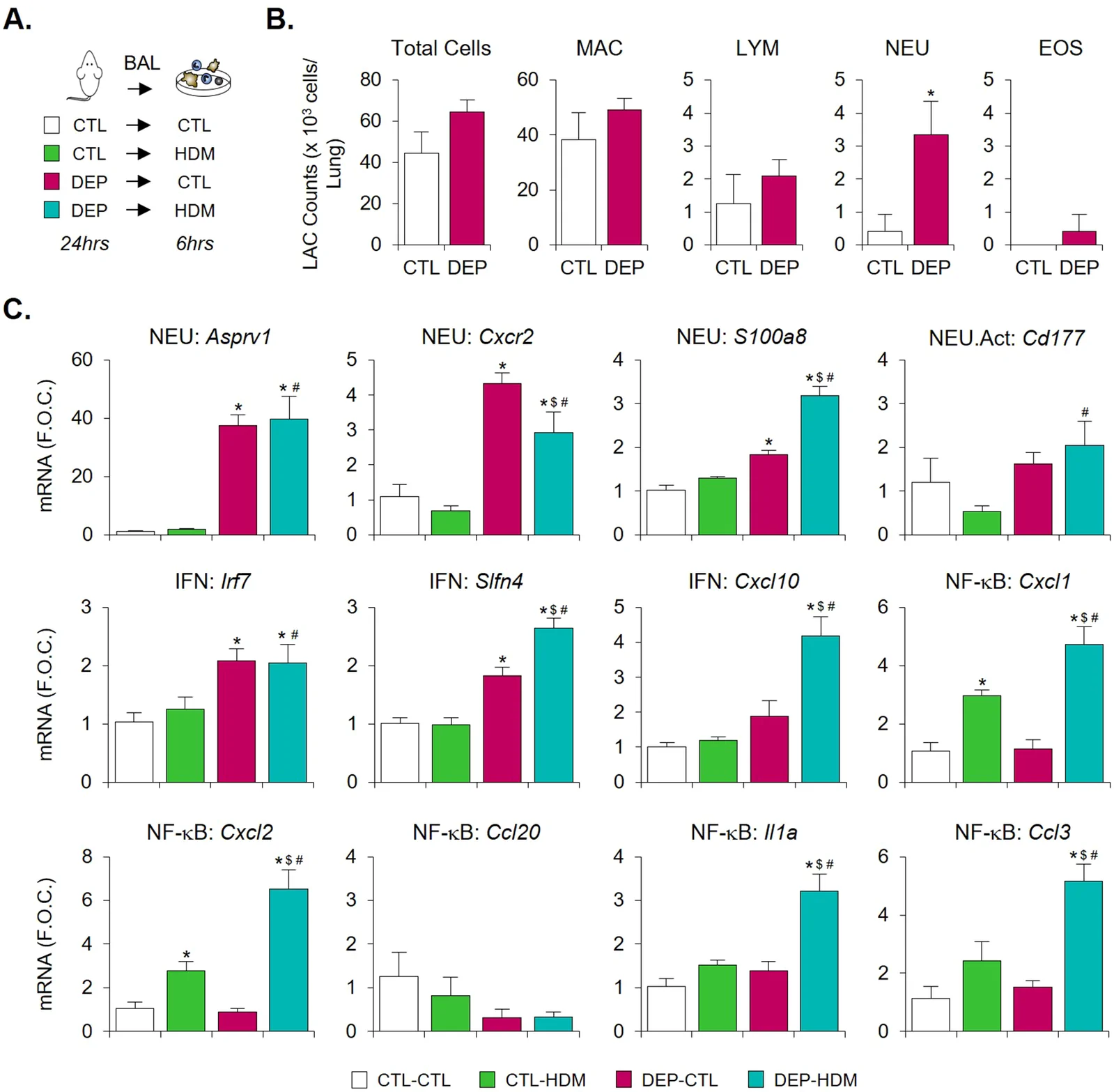
DEP pre-treatment enhances LAC ex vivo inflammatory responsiveness to HDM. Mice (n=4 per group) were intranasally exposed to PBS (CTL) or DEP (2.5 mg/kg) for 24 hrs followed by removal of LAC by bronchoalveolar lavage (BAL) and *ex vivo* exposure to either CTL or HDM (25 µg/ml) for a further 6 hrs **(A)**. Cells were examined for total **(B)** or differential cell population (MAC; Macrophages, LYM; Lymphocytes, NEU; Neutrophils, EOS; Eosinophils) counts using microscopic analysis **(B)**. mRNA was isolated and analysed for neutrophil marker (NEU: Asprv1, Cxcr2, S100a8, Cd177) or inflammatory gene expression by RT-PCR **(C)**. Fold over control (CTL-CTL) levels (F.O.C.) were calculated and results expressed as Mean +/- SEM for each treatment group. Statistically significance was calculated using ANOVA with Fischer LSD test and indicated as * (p<0.05 v CTL-CTL), $ (p<0.05 DEP-HDM v DEP-CTL) and # (p<0.05 DEP-HDM v CTL-HDM).

**Figure 7:**
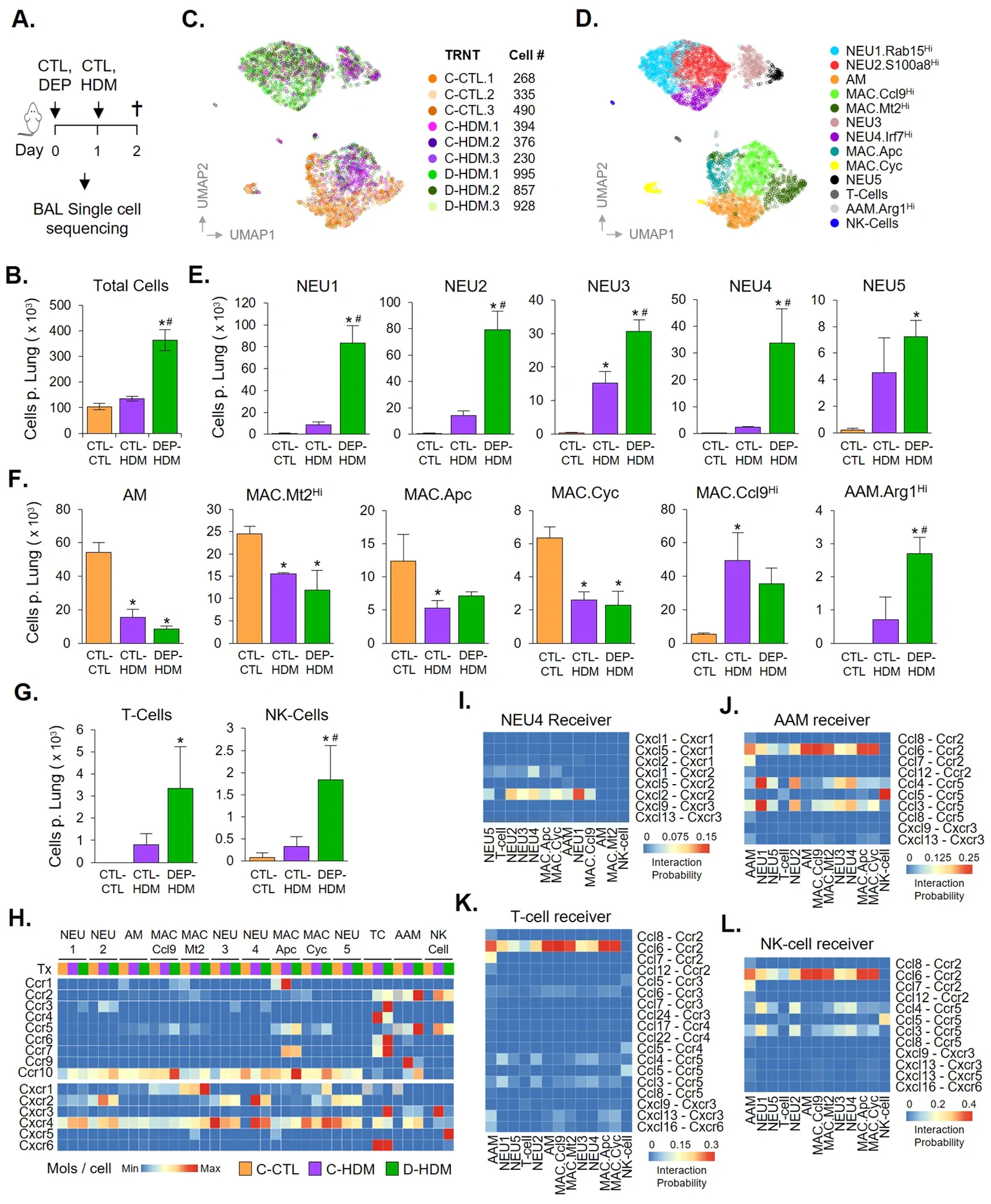
A single exposure to DEP alters the cellular and transcriptional landscape of the LAC in response to HDM. Mice (n=3 per group) were intranasally exposed to a single treatment of PBS (CTL) or DEP (2.5 mg/kg) for 24 hrs followed by a single exposure to HDM (1.25 mg/kg) for 24 hrs **(A)**. Total cell counts from BAL were determined using microscopy **(B)**. LAC derived cells were examined for cell type marker and inflammatory gene expression using TGE ScSeq analysis **(C–L)** with levels in individual mice identified within the dataset using antibody-based sample tags. ScSeq data was visualised as UMAP plots of cells per mouse/treatment group **(C)** or cell type **(D)**. LAC cell counts normalised for total lung content are displayed **(E-G)**. Expression levels for chemokine receptor mRNA across cell types and treatment (Tx) are displayed as normalised expression to maximum for each gene **(H)**. CellChat analysis was used to identify ligand – receptor interaction pairs, with strength of association indicated as interaction probability for NEU, AAM, T-cell & NK-cell receiver cell populations **(I-L)**. Statistical comparisons used ANOVA with Fisher LSD test (* = <0.05 compared to CTL-CTL. # = <0.05 D-HDM v C-HDM). All annotated populations were included in CellChat inference. Panel I displays NEU4 as the receiver population.

**Figure 8:**
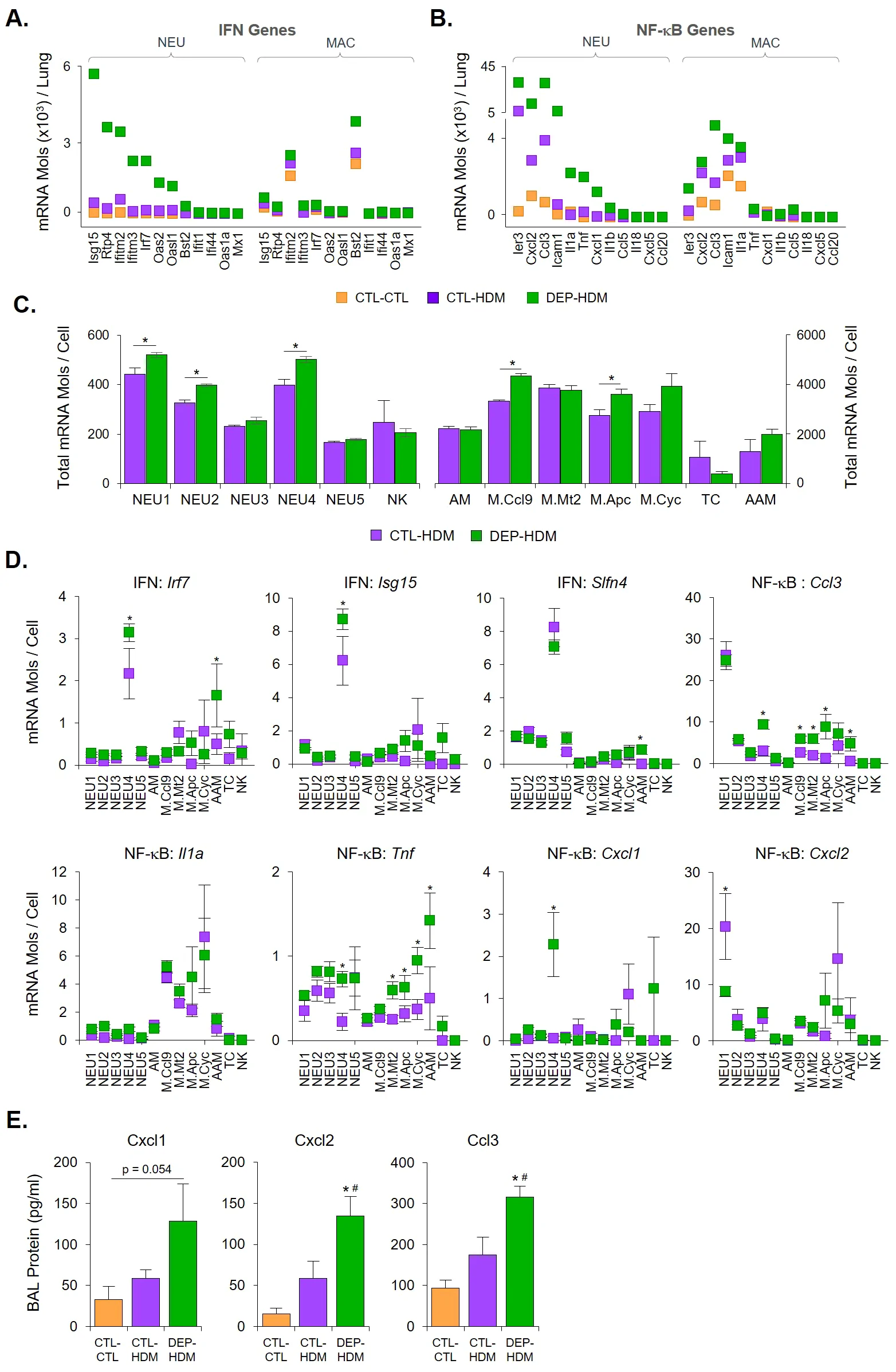
LAC neutrophil and macrophage sub-populations display differential inflammatory responses to HDM after DEP priming. Single cell mRNA sequencing data from **Figure 7** was further interrogated for inflammatory gene expression alterations. Total LAC mRNA molecules per lung for IFN **(A)** and NF-κB **(B)** dependent genes are displayed as the sum of all NEU and MAC sub-population counts. Total mRNA molecules per cell were also displayed **(C)**. mRNA molecules per cell for individual inflammatory genes are indicated **(D)**. BAL protein levels for Cxcl1, Cxcl2 and Ccl3 were examined using ELISA and are presented as fold over control (F.O.C) **(E)**. Results are expressed as mean +/- SEM with statistical comparison between CTL-HDM v DEP-HDM groups carried out using student T-test (* indicating p<0.05). For ELISA, ANOVA with Fisher LSD test (* = <0.05 compared to CTL-CTL. # = DEP-HDM v CTL-HDM).

Four related exposure designs were used. For repeat priming experiments in **Figures 1**, **2**, **3**, **4**, mice received PBS or DEP on days 1, 3, 6, 8, 10, 13, 15 and 17 followed by PBS or HDM on day 20 and were sampled on day 21. For the repeat DEP single cell experiment in **Figure 5**, BAL was collected 12hr after the eighth PBS or DEP exposure. For the ex vivo experiment in **Figure 6**, BAL cells were collected 24hr after a single PBS or DEP exposure and stimulated ex vivo with PBS or HDM for 6 h. For the single dose sequential experiment in **Figures 7**, **8**, mice received PBS or DEP followed by PBS or HDM 24hr later and were sampled after a further 24hr.

### mRNA isolation

25mg of lung tissue (post BAL procedure) was homogenised in 350µl RLT buffer (RNeasy mini kit from Qiagen; Cat # 74134) using MagNA lyser bead disruption (Roche Diagnostics, West Sussex, UK) and passed through a QIAShredder column (Qiagen; Cat # 79656). BAL immune cells were directly lysed into RLT buffer. Total RNA for tissue and BAL cells was isolated using the RNeasy mini kit (Qiagen, Valencia, CA) according to the manufacturers protocol. RNA quantity and purity were determined using nanodrop (ThermoFisher Scientific, UK) and those samples with A260/A280 and A260/A230 ratios of between 1.8 – 2.2 and 1.5 – 2.2 respectively were selected to proceed with bulk-seq or RT-PCR analysis.

### Bulk seq and pathway analysis

Total RNA from Lung tissue (TISS) or luminal airway cells (LAC) isolated from BAL, were analysed for integrity and purity using an Agilent 2100 Bioanalyzer. Samples with an RNA integrity number (RIN) above 7.0 were processed for bulk-seq analysis. cDNA libraries underwent paired end (150PE) sequencing for transcriptomic analysis using the DNBseq platform (BGI Genomics, HK). 20 million clean reads for each sample were trimmed to remove remaining adapters and other variable sequences, followed by gene level annotation using the Mm39 reference genome builds using CLC Genomics Workbench software (CLCBIO, Aarhus, Denmark). CLCBIO software was also used to perform differential expression using a general linearised model with a negative binomial distribution. For comparative analysis of select genes between treatment groups data was also extracted as reads per kilobase per million (RPKM). Pathway analysis was used to categorise differentially expressed genes according to cellular signalling associated (hallmark.MySigDB) and transcription factor regulated (TF.Target.RegNetwork) databases, using iDEP software using a GSEA pre-ranked method (https://bioinformatics.sdstate.edu/idep/). Differentially expressed gene input lists were ranked based on fold change (>2) and FDR (<0.1). Groups of cell type specific genes were used for cell type interpolation of bulk-seq data and were collated through manual curation of the literature, the Immgen database and a mouse lung cell atlas (39–42) as previously described (43). Relative quantification of cell type levels from bulk-seq was then calculated using an enrichment score based method (44, 45) as the average differential expression score (DES) (Log2(FC) x -Log10(FDR q Value)) across genes within each cell type group.

### Single cell mRNA sequencing

Bronchoalveolar lavage derived LAC cells were processed for single cell analysis using a targeted gene expression (TGE) profiling method using the Rhapsody express system and scanner (BD Biosciences, UK). 397 genes from the mouse immune cell panel (Cat# 633753, BD Biosciences UK) (**Supplementary Table 1**) and 93 genes from a custom designed panel (**Supplementary Table 2**) were used for TGE profiling. Multiplexing of samples was carried out using Sample-tag antibodies (Cat# 633793). Procedures were carried out according to the manufacturer’s instructions with ~ 2 x 10^5^ LAC cells starting material for each sample. Briefly, non-specific Fc Receptor sites were blocked with Mouse BD Fc Block™ prior to Sample tag labelling for 20 minutes at room temperature. After washing and equal proportion sample pooling, cells were stained with viability markers Calcein-AM and DRAQ7 and counted using the Rhapsody scanner. Cells were passed through a 37μm cell filter and between 5000 – 10000 viable cells were loaded onto each cartridge. After cell lysis and bead capture of mRNA and multiplexing oligonucleotide tags, on bead cDNA synthesis was carried out according to manufacturer’s guidelines (Cat# 633773). Purification of cDNA and Sample-tag PCR products was carried out using AMPure XP magnetic beads (Beckman Coulter, cat#A63881). Index library preparation was performed with 2.5ng/μL TGE cDNA and 1ng/μL for Sample-tag PCR products using a further 6 PCR cycles of amplification. cDNA was quantified using Qubit and Agilent Tapestation and pooled (~95/5% mRNA/Sample-tag ratio) to achieve a final concentration of 5 nM. Libraries were spiked with 15% PhiX to increase sequence complexity and sequenced (100PE) on a HiSeq X-Ten sequencer (Illumina). FastQ output files were annotated to reference libraries to identify cell label and unique molecular identifier (UMI) sequences using the BD Rhapsody™ Analysis Pipeline Version 1.2 (BD Biosciences, UK). The number of distribution-based error correction (DBEC) molecules (mols) per cell was then extracted, multiplets and undetermined Sample Tag cells removed. Quality control, removal of low-quality cells and low expressed genes, as well as normalisation (10,000 mols per cell) was carried out using SeqGeq version 1.7.0 (FlowJo LLC, US) software. Immune cell populations were identified using Seurat 3.0 plugin-based clustering and visualised using UMAP dimensionality reduction of highly dispersed expressed genes. We inferred intercellular communication networks from single-cell RNA-seq data using the R package CellChat. Cells were grouped by annotated cell type, and ligand–receptor interactions were identified using the built-in mouse database. Communication probabilities were computed from normalized gene expression and aggregated to summarize interaction strength and signalling pairs between cell groups. Results were output as heatmaps.

### ELISA

Cleared BAL fluid protein levels for Cxcl1 (Cat# DY453), Cxcl2 (Cat# DY452), Cxcl5 (Cat# DY443), Ccl3 (Cat# DY450), Ccl6 (Cat# DY487), Ccl20 (Cat# DY760) and Cxcl10 (Cat# DY466) using DuoSet ELISA kits from Bio-Techne (Abingdon, UK). Plasma total IgE concentrations were analyzed using a BD OptEIA ELISA kit (cat# 555248; BD Bioscience, Oxford, UK). All procedures were carried out according to the manufacturer’s instructions. Samples were analysed in duplicate. Absorbance was measured at 450 nm with background levels at 570 nm using a plate reader (Bio-Tek Synergy HT). Extrapolation of protein levels was carried out from a standard curve of recombinant proteins.

### Ex vivo culture and RT-PCR analysis

LAC were isolated using bronchoalveolar lavage from mice primed with DEP or PBS intranasal administration for 24 hrs. Cells were resuspended in RPMI media containing 0.1%(w/v) human serum albumin and penicillin/streptomycin (100U/mL) and plated at a concentration of 1x10^4^ cells/well onto low attachment 96 well plates (Cat# CLS3474, Merck). Cells were immediately treated with PBS (CTL) or HDM (Greer Stallergenes) for 6 hrs before mRNA isolation and PCR analysis. Total RNA was isolated using the RNeasy mini kit, as described previously and cDNA synthesised using a random hexamer-based protocol (Cat# EP0741, ThermoFisher Scientific, UK). SYBR green based quantitative real time PCR (40 cycles) was carried out using the QuantStudio™ 6 Flex PCR System. Gene specific primers were obtained from the published literature or designed using Primer 3 software and supplied by Integrated DNA technologies, UK (**Supplementary Table 3**). Fold change in mRNA expression was assessed using the delta-delta Ct method with normalization to the housekeeping gene Hprt1.

### Statistical analysis

Statistical significance between groups was carried out with Graphpad Prism Software version 9 using one-way ANOVA/Fisher’s LSD Test unless otherwise stated. Results are expressed as mean ± standard error of the mean (SEM).

## Results

### DEP priming enhances HDM recruitment of neutrophils to the airway lumen

To investigate the ability of DEP pre-exposure to modify the pulmonary inflammatory response to subsequent HDM exposure, we exposed mice to repeated DEP administration over 20 days followed by a single HDM exposure for 24 hrs (**Figure 1A**). A repeat exposure protocol was used to create a stable DEP conditioned lung environment, while also minimising any DEP induced acute inflammatory interactions with HDM and possible contribution of effects attributable to HDM co-exposure mechanisms. A single exposure to HDM after repeated saline exposure (C-HDM) resulted in increased total BAL cell counts. Repeated DEP exposure followed by a single saline exposure (D-CTL) also demonstrated increased total BAL cell counts, at levels higher than that observed with C-HDM. DEP priming followed by HDM for 24hrs resulted in significantly higher levels of total BAL cells, when compared to both D-CTL and C-HDM (**Figure 1B**). Serum IgE was used as systemic indicator of type 2 inflammatory skewing as a potential conditioning outcome from repeat DEP exposure (**Figure 1C**). No significant changes were observed, except for a modest non-significant increase with D-CTL exposure. Differential counts for broad immune cell types were next investigated for the retrieved BAL cells (**Figure 1D**). Macrophage (MAC) cell numbers per lung were increased with C-HDM and D-CTL. D-HDM also increased MAC levels compared to control C-CTL and C-HDM but were significantly reduced when compared to D-CTL levels. Total lymphocyte (LYM) levels were increased with D-CTL but unmodified by HDM exposure (D-HDM). While DEP conditioning (D-CTL) produced a non-significant increase in BAL neutrophils (NEU), this was significantly increased with subsequent HDM exposure (D-HDM), an effect that was also significantly higher than that induced by DEP unprimed single HDM exposure (C-HDM) (**Figure 1D**). No significant changes in eosinophils were observed (**Figure 1D**). The absence of a significant systemic IgE or eosinophil response is consistent with the acute, unsensitised single HDM exposure design, which was intended to capture early innate events rather than a mature adaptive allergic response. Comparison of total cell counts to cell type specific counts reveal different patterns of responses. The additional total cell signal in D-HDM v D-CTL, was accounted for primarily by the marked neutrophil increase, which was partly offset by lower macrophage numbers, while broad lymphocyte counts were not further increased.

### DEP priming enhances HDM induced inflammatory transcriptional programs within the lung tissue compartment

Bulk RNA-seq was next used to interrogate the lung tissue (TISS) samples post lavage, for alterations in inflammatory transcriptional programs and cell type specific responses, associated with the DEP priming effects on HDM responsiveness. Baseline HDM responses in control primed lung tissue (C-HDM) were initially characterised (**Figures 2A, B**). This identified predominantly upregulated genes (**Figure 2A**), categorised as interferon and NF-κB regulated by pathway analysis (**Figure 2B**). DEP primed (D-CTL) lung tissue was also examined for transcriptional alterations that may contribute to altered responsiveness from a subsequent HDM exposure (**Figures 2C, D**). This identified pro-inflammatory NF-κB dependent genes such as Cxcl1, Cxcl5 and Ccl20 (**Figure 2C**) as upregulated, supported by pathway analysis for this transcription factors involvement (**Figure 2D**). No indication of IFN dependent gene regulation was found with D-CTL treatment. A comparison of D-HDM to C-HDM groups directly revealed an increase in predominantly immune associated gene expression (**Figure 2E**), classified more broadly as IFN and NF-κB regulated by pathway analysis (**Figure 2F**). As the latter comparison includes genes that will also be altered by DEP alone, and not necessarily responsive to HDM stimulation, to specifically identify enhanced HDM responses from DEP priming, we also examined those statistically significant regulated genes that were present in both D-HDM v C-HDM and D-HDM v D-CTL individual comparisons (**Figure 2G**). This refined group was dominated by immune associated genes, again including IFN and NF-κB upregulated. Exemplar IFN regulated gene expression (Irf7, Isg15, Ifit1) followed a pattern of enhanced HDM responsiveness with DEP priming but no alteration by DEP alone (**Figure 2I**; upper panels). NF-κB pathway master regulators Il1a, Il1b and Tnfa expression followed a similar pattern (**Figure 2I**; middle panels).

Cell interpolation analysis was used to infer cell type changes from the bulk-seq data (**Figure 2H**). DEP primed (D-CTL) lung tissue displayed modest changes in cell types compared to HDM exposed groups. DEP priming resulted in HDM induced increases in a number of cell types including recruited macrophage cell types recently identified as increased after DEP and HDM co-exposure (43). These cells display overlapping profiles for interstitial and alternatively activated macrophages, which were also increased by HDM with DEP priming. Conventional dendritic cells, previously categorised as expressing IFN genes (46) were also increased by HDM with DEP priming. The final group of cells identified as enhanced by DEP priming were NEU (**Figure 2H**), consistent with observations of enhanced levels of these cells in BAL cell counts (**Figure 1D**). Expression of the neutrophil marker Asprv1 and the activated neutrophil marker Cd177 were significantly higher with D-HDM than other treatment groups, with magnitude of increase much higher for Cd177 (**Figure 2I**; lower panels). An analysis of genes that were proportionally increased, decreased or unaltered by DEP priming was also carried out (**Supplementary Figures 1A–G**). This method mainly categorised genes dependent on whether D-CTL levels were altered or remained unchanged.

### DEP priming enhances HDM induced inflammatory transcriptional programs within the luminal airway compartment

Analysis of transcriptional changes in the LAC compartment were initially examined for baseline HDM exposure in control primed samples (C-HDM). A robust response compared to C-CTL with the majority of changes upregulated genes involved in inflammatory processes (**Figure 3A**). Pathway analysis revealed three main categories of gene classifications as most significant (**Figure 3B**) similar to changes in matched TISS samples; IFN and NF-κB regulated genes as well as genes involved in cell cycle regulation (E2F and Mcm signalling). DEP priming effects (D-CTL) within the LAC was also examined and similar to C-HDM effects, the majority of gene expression changes were upregulated rather than downregulated (**Figure 3C**). Pathway analysis of D-CTL regulated genes resulted in cell cycle (E2F, G2M, Mcm) categorisation as most prominent followed by inflammatory responses such as NF-κB and TNF signalling (**Figure 3D**). Comparison of D-HDM to C-HDM transcriptomic differences to determine LAC enhanced HDM response from DEP priming are displayed in **Figure 3E**. Through pathway analysis inflammatory processes dominated by IFN and NF-κB as well as cell cycle signalling were most significantly enhanced (**Figure 3F**). To remove DEP alone contributions and to specifically identify true HDM enhanced gene expression, a further refined gene list was made using only those genes that were statistically significantly different in both D-HDM v C-HDM and D-HDM v D-CTL comparisons. This restricted list for LAC genes (**Figure 3G**) contained 402 genes of which there were clear exemplars for IFN (Irf7, Cxcl10, Isg15, Cxcl11) and NF-κB (Il1a, Il1b, Tnf) pathway activation. Examination of expression patterns in more detail revealed that IFN responsive genes were robustly HDM induced in control unprimed lungs, which was further enhanced by DEP priming, while DEP priming itself (D-CTL) did not significantly alter expression. This pattern was also observed for Il1b, with Il1a and Tnf expression only significantly upregulated by HDM under DEP primed conditions (**Figure 3I**). Cell cycle responsive pathways (E2F, G2M, Mcm) were also identified as upregulated with HDM and DEP treatments (**Figures 3B, D**). Examination of prototypical cell cycle gene expression changes in the LAC compartment revealed an increase in Pclaf with HDM treatment and DEP treatment separately, which was further increased on combination (**Supplementary Figure 2B**), an effect also observed within the TISS compartment (**Supplementary Figure 2A**). Similar effects were also observed for the cell cycle gene Top2a. Similar to analysis carried out for TISS samples, examination of genes that were proportionally increased, decreased or unaltered by DEP priming in LAC was also carried out (**Supplementary Figures 1H–N**). Again, this method mainly categorised genes dependent on whether D-CTL levels were altered or remained unchanged. Interestingly, we categorised the majority of HDM inducible IFN genes within the proportionally unenhanced category (**Supplementary Figures 1I, J**), indicating that some intrinsic change to the lung occurs (e.g. responsive cell type recruitment or resident cell sensitivity changes), which explains enhanced HDM effects. A similar explanation may also account for DEP enhanced HDM responsive genes (**Supplementary Figures 1K, L**).

Cell interpolation was carried out on the LAC bulk-seq data (**Figure 3H**). DEP priming (D-CTL) resulted in an increase in a number of cell types including AM.Cyc, RM.Gp, AAM, cDC2, NEU, T-cells CD4/TH2 T-cells, gdT and B-cells, all potential facilitators of increased HDM responsiveness. HDM treatment in unprimed lungs (C-HDM) also resulted in increased signals for immune cell types, most prominent for cDC2-IFN, MON.c, NEU and NK cells. Priming with DEP resulted in enhanced HDM responsiveness (D-HDM) with RM.Gp1, NEU and NK cells all displaying increased levels compared to HDM alone induced levels. Interestingly the B-cell response induced by D-CTL was reduced with D-HDM exposure. Examination of the canonical Neutrophil marker Asprv1 revealed modestly enhanced levels in D-HDM compared to either C-HDM or D-CTL, with levels suggesting an additive effect of co-treatment (**Figure 3I**). Levels of the activated neutrophil marker Cd177 did not show increased HDM responsiveness with DEP pre-treatment (**Figure 3I**) in contrast to what was observed in the TISS compartment (**Figure 2I**). Post lavage TISS contains vascular, interstitial and tissue adherent cells, whereas LAC contains cells recovered from the airway lumen. The compartment difference may therefore reflect location and transmigration associated neutrophil activation states rather than a global activation, particularly because the activated neutrophil marker Clec4d increased in both compartments (**Supplementary Figures 2A, B**). A similar enhanced effect was also demonstrated for classical monocyte markers (Ly6c2, Plac8) in the LAC (**Supplementary Figure 2B**), a trend also observed but not statistically in TISS samples (**Supplementary Figure 2A**). The most prominent enhanced effect of DEP priming on HDM induced cell type markers was for CD8 (Cd8a, Cd8b1; **Supplementary Figure 2B**) and NK cells (Nrc1; **Figure 3I**), an effect not observed within the TISS compartment analysis.

### Direct comparison of LAC and TISS for compartment dependent transcriptional changes to HDM on DEP priming

The next set of analysis directly compared TISS and LAC bulk RNA-seq data for compartment specific expression of HDM induced genes enhanced by DEP priming (**Figure 4**). Those genes which were statistically significantly altered in both D-HDM v C-HDM and also D-HDM v D-CTL comparisons were selected. DES levels for these genes as compared to C-CTL, were calculated for both TISS and BAL samples and plotted as the difference in scores between compartments (**Figure 4A**). This revealed different patterns of enhanced genes across compartments (**Supplementary Table 4**). Upregulated genes were selected as TISS or LAC dominant expression or differentially expressed at the same level across compartments (Common). These genes were categorised as IFN and NF-κB dependent, as well as for cell type specific markers (**Figure 4B**). The TISS compartment displayed a greater number of NF-κB dependent genes than the LAC compartment, with a common set of NF-κB genes also identified. The majority of IFN dependent genes were identified in the LAC and Common compartment compared to TISS. Activated neutrophil markers were expressed in the TISS while more canonical markers (E.g. Asprv1) were identified within the LAC compartment. NK and CD8 cells typically share some exclusive gene markers (e.g. Nkg7 and Gzma). These and the NK specific marker Ncr1 were exclusively expressed within the LAC compartment. Similar categorisation of gene expression was also found with other treatment comparisons within the data (**Supplementary Figures 3A–D**). Protein levels in BAL fluid for the chemokines, Cxcl1, Cxcl2, Ccl20, Cxcl10, Ccl2 and Ccl3 were examined. All displayed increased levels of expression in DEP primed (D-HDM) when compared to control (C-CTL) and enhanced levels above unprimed (C-HDM) treatments (**Figure 4C**).

### Single cell sequencing analysis of DEP primed changes within the LAC

The LAC compartment was further investigated for immune cell type specific alterations from DEP priming, that would provide insight into how increased responsiveness of the lung to HDM occurs. This was carried out using a single cell mRNA sequencing approach focussed to immune cell markers and inflammation associated genes (**Figure 5**). Mice were subject to repeated DEP pulmonary exposure, with LAC isolated at 12 hrs after the last exposure (**Figure 5A**) to capture both alterations in the cellular landscape of the DEP primed environment, as well as any active cellular recruitment mechanisms. Total cell counts revealed an increased number with DEP treatment (**Figure 5B**). A total of 10 distinct cell types were identified through Seurat based clustering of gene markers (**Figure 5D**, **Supplementary Figure 4A**). There was a decreased number of LAC cells per lung for alveolar macrophages (AM) and antigen presenting macrophages (MAC.Apc), while no change in Mt2 expressing macrophages (MAC.Mt2) or Irf7 expressing macrophages (MAC.Irf7). All other cell types were increased with DEP repeat exposure, with the magnitude of neutrophil (NEU) cell recruitment to the LAC compartment arguably the greatest (**Figure 5E**). Examination IFN and NF-κB dependent gene expression, previously identified as enhanced with HDM exposure, for levels of expression in DEP primed LAC was next investigated (**Figure 5F**). For the IFN pathway gene Irf7, expression was found predominantly in MAC.Irf7 cells, while for Slfn4, expression was exclusive to NEU. Cxcl10 is predominantly an IFN dependent gene but can be induced through NF-κB activation also (47) and was increased with DEP treatment within MAC.Cyc and MAC.Irf7 cells (**Figure 5F**). The NF-κB dependent chemokine genes Cxcl1 and Cxcl2 were modestly increased within MAC.Mt2 cells in DEP primed lungs, while the largest increase in expression of Cxcl2 was found in NEU (**Figure 5F**). DEP induced increases in the NF-κB master regulator gene Il1a were not observed within NEU but across different MAC populations.

The recruitment of immune cells to the lung is governed largely by local chemokine expression and by the selective expression of cognate chemokine receptors on distinct immune-cell subsets (48, 49). Examination of CKR mRNA expression within LAC of DEP primed lungs was examined for cell type specific expression and demonstrated increased levels of Ccr1 and Cxcr4 in the NEU population (**Figure 5G**). There was also strong and exclusive expression of Cxcr2 in the NEU population, a CKR known to mediate recruitment of NEU to the lung (48). Cell communication inference analysis was also carried out to examine which cell types within the LAC have the potential to interact through ligand receptor matching (**Supplementary Figure 4B**). A focus to those chemokine (CK) signals responsible for recruitment of NEU revealed a number of CK and CKR pairs of interest (**Figure 5H**). Ccr1 expression was identified as having a strong interaction score with a number of potential ligands derived mainly from macrophage populations. Due to the wide-ranging expression of Ccr1 on different cell types in both NEU and MAC populations, it’s specificity for being responsible for recruitment of NEU to the lung is unclear. Cxcr2 on the other hand is more likely to direct NEU to the lungs and was identified as having significant interaction with Cxcl2 derived from NEU and MAC.Mt2 cells. A similar but less significant interaction was observed for Cxcl1 expressed in MAC.Mt2 cells (**Figure 5H**). Examination of protein levels for chemokines in BAL from these lungs revealed increased levels of Cxcl1, Cxcl2 and Cxcl5 but not Ccl6 with DEP priming compared to CTL exposures (**Figure 5I**).

### A single DEP exposure increases NEU recruitment to the LAC and transcriptional responsiveness to HDM ex vivo

We next examined the effect of a single exposure to DEP has on the recruitment of immune cells to the LAC compartment, and whether these cells display altered HDM responsiveness when exposed outside of the lung environment, when cultured *in vitro* (**Figure 6A**). Marginal increases in total, MAC, LYM and EOS cell counts were observed after 24 hr DEP exposure, but these were not statistically significant (**Figure 6B**). NEU levels were however significantly increased (**Figure 6B**). This was supported by increased levels of NEU markers Asprv1, Cxcr2 and S100a8 gene expression in DEP-CTL exposures (**Figure 6C**). The levels of Asprv1 did not change upon ex vivo exposure to HDM for 6 hrs, while levels of Cxcr2 and S100a8 were down and upregulated respectively. Interferon (IFN) associated genes also displayed differential regulation. Irf7 was increased by DEP-CTL but not further altered by HDM exposure (DEP-HDM). Alternatively, DEP-CTL induced levels of Slfn4 and Cxcl10 were further increased by HDM exposure (**Figure 6C**). NF-κB dependent genes Cxcl1 and Cxcl2 were not altered in DEP LAC but demonstrated *ex vivo* HDM responsiveness (CTL-HDM), which was further increased in DEP primed cells. This enhanced effect of DEP priming was also observed for the NF-κB master regulator Il1a and the Ccr5 chemokine receptor ligand Ccl3. The differential regulation of genes observed here indicate increased NEU recruitment with DEP as a potential mechanism for enhanced HDM responsiveness in inflammatory gene expression.

### Single cell sequencing analysis of HDM transcriptional responsiveness after a single DEP exposure

Having demonstrated a single DEP exposure increases NEU recruitment to the LAC compartment, and enhanced HDM responsiveness ex vivo, we next examined the cell types responsible for this effect using ScSeq. Mice were exposed to DEP for 24 hrs followed by HDM for a further 24 hrs (**Figure 7A**) and demonstrated an increase in total cell counts in DEP primed HDM induced levels (DEP-HDM) compared to unprimed controls (CTL-HDM) (**Figure 7B**). A total of 13 different cell types were identified through Seurat based clustering of gene markers (**Figure 7D**, **Supplementary Figure 5A**). Five different sub-populations of NEU were identified, all but NEU5 were significantly up-regulated in DEP-HDM versus CTL-HDM treatments (**Figure 7E**). There was a shift in control macrophage populations present in CTL-CTL untreated mice towards the MAC.Ccl9Hi population by HDM treatment, which were not further altered by DEP pre-treatment (**Figure 7F**). Alternatively activated macrophages (AAM.Arg1Hi) increased by HDM, were further enhanced by DEP pre-treatment (**Figure 7F**). These AAM cells were not induced by repeat DEP treatment alone (**Figure 5**, **Supplementary Figure 4A**). T-cells and NK-cells were also increased with HDM treatment only when DEP was previously administered (**Figure 7G**). CellChat cell communication inference analysis, including all annotated cell populations, was used to explore recruitment signals governing enhanced HDM recruitment of cells to the LAC by analysing DEP-HDM exposure samples (**Figures 7I–L**, **Supplementary Figure 5B**). Receiver focused visualisation emphasised NEU4 because this Irf7 high subset displayed the most selective Cxcr2 expression and a strong per cell inflammatory increase. NEU5 was the only neutrophil subset not significantly increased in DEP-HDM versus CTL-HDM. Neutrophils, especially NEU4 displayed specific expression of Cxcr2 (**Figure 7H**) and significant interaction was found for Cxcl2 derived mainly from NEU and non-AM macrophages (**Figure 7I**). A similar interaction was found for Cxcl1, derived most strongly from the same NEU4 cell population. Significant interactions were also found for Ccr2 and Ccr5 across AAM, T-cell and NK-cell receiver populations implicating chemokines such as Ccl6, Ccl3, Ccl4 and Ccl5 (**Figures 7J–L**) as potential recruitment signals. Indeed, Ccr2 and Ccr5 were expressed selectively in AAM, T-cell and NK-cells compared to other cell types (**Figure 7H**), indicating a potential specific mechanism of cellular recruitment. A similar expression profile was observed for the Cxcr3 receptor across these cell types and ligands including Cxcl9 and Cxcl13 were identified as potential mediators of recruitment on CellChat analysis.

IFN and NF-kB dependent genes identified as enhanced on bulk-seq analysis from repeat DEP primed exposure experiments (**Figure 2**, **3**) were analysed for LAC cell type specific expression levels (mRNA molecules per lung) after HDM treatment in single dose DEP primed and unprimed treatments (**Figure 8A**, **Supplementary Figures 6A–D**). IFN genes for the most part displayed enhanced HDM responsiveness in DEP primed exposure samples in NEU, except for two genes Ifitm2 and Bst2 in macrophages where CTL levels were high. NF-κB dependent genes also displayed the strongest enhanced response in neutrophils compared to macrophages, but the number of genes increased was greater than seen with IFN genes. The magnitude of HDM induced increase in mRNA levels with DEP priming was greater in neutrophils than macrophages.

Further interrogation of this HDM responsive LAC Scseq dataset was carried out to determine whether increases in total mRNA molecules per lung could be explained by either increased levels of transcript within each cell type or a consequence of increased numbers of cells in the LAC, or a combination of both (**Figures 8C, D**). Total mRNA levels per cell, from ~490 inflammatory genes within the TGE Scseq immune cell panel, were calculated for CTL-HDM and DEP-HDM populations (**Figure 8C**) and revealed significant increases in levels with DEP priming in NEU1, NEU2 and NEU4 neutrophil populations. There was also a significant increase in levels in M.Ccl9 and M.Apc macrophage populations. These changes indicate increased inflammatory responsiveness within cells, as well as increased levels of inflammatory cells contribute to increased HDM transcriptional responses in the LAC from DEP priming. Examination of a selection of IFN dependent genes indicates that the Irf7^HI^ NEU4 sub-population is a major source of IFN pathway responsiveness in the LAC (**Figure 8D**). Observations with NF-κB dependent genes are more mixed, with incidents of macrophage predominant expression (Il1a) or similar levels of expression across neutrophils and macrophages (E.g. Tnf, Cxcl2). Interestingly DEP-HDM Cxcl1 mRNA levels per cell were increased in NEU4 compared to CTL-HDM, while the other neutrophil chemokine receptor (Cxcr2) ligand Cxcl2 displayed less mols per cell. Importantly, the chemokine Ccl3 displayed nearly 3-fold higher mRNA levels per cell in NEU4 for DEP-HDM compared to CTL-HDM. No other NEU cell population displayed increased levels of Ccl3 with DEP priming, while all MAC subpopulations except AM did (**Figure 8D**). Finally, we examined protein levels of Cxcl1, Cxcl2 and Ccl3 expression in the BAL fluid and found that CTL-HDM increased levels of each chemokine compared to CTL-CTL, which was further enhanced by DEP priming (DEP-HDM) (**Figure 8E**).

## Discussion

This study was designed to determine whether pollutant particle exposure can prime the lung such that the acute response to a later aeroallergen exposure is fundamentally altered, while explicitly minimizing direct particle–allergen physical interaction and pre-existing allergen sensitization associated inflammatory microenvironments, two common confounders in mixed exposure models. By separating DEP exposure from an HDM challenge by 24 hours in the single dose study and by 72hr after the final DEP exposure in the repeat dose study, we aimed to isolate priming mechanisms that operate through changes in lung cellular composition, activation thresholds, and intercellular signalling rather than through coincident physicochemical interactions at the time of allergen delivery. Across both the single and repeat DEP conditioning approaches, the most prominent priming outcome was amplified leukocyte recruitment to the airway lumen, characterized primarily by increased neutrophils. This finding is notable because acute allergen responses are commonly interpreted through a lens of type 2 (T2) inflammation context, whereas our sequential exposure design identified a strong neutrophil component at the acute time scale. This may be relevant for particular types of asthma development and exacerbation events, as neutrophilic airway inflammation is widely associated with severe disease features and reduced corticosteroid responsiveness (32, 33, 50). Even beyond neutrophilic dominant asthma endotypes, neutrophils can mechanistically participate in pollutant allergen exacerbation biology (34) and can adopt local effector states that may contribute to allergic airway pathology (51). The lack of significant neutrophilia in the D-CTL group in **Figure 1** and the acute increase in **Figure 6** is therefore not directly at odds. **Figure 1** was sampled 96hr after the final repeated DEP dose, whereas **Figure 6** was sampled 24 h after one DEP dose, and **Figure 5** also demonstrated recruitment 12hr after the final repeat dose. These data are consistent with an acute neutrophil influx that wanes while a primed inflammatory state persists.

A central insight finding from bulk transcriptomics is that DEP priming establishes an inflammatory response that is qualitatively distinct from, yet synergistic with, baseline HDM induced programs. DEP conditioning predominantly enriched NF-κB associated chemokines and inflammatory mediators, whereas HDM induced both NF-κB and interferon associated transcriptional programs that were further amplified when HDM occurred in a DEP primed lung. This pattern is biologically plausible for HDM because HDM extracts contain microbial associated ligands that can engage multiple innate recognition pathways and diversify acute signalling, including interferon linked pathways (52). The interferon stimulated gene (ISG) signatures induced by HDM in our study also align with clinically relevant transcriptional phenotypes. In human airway datasets, type I interferon/ISG programs have been associated with reduced lung function in asthma, independent of viral infection and independent of T2 inflammation (53). They are also associated with neutrophilic but not eosinophilic asthma (54) and can inhibit aspects of T2 development in experimental contexts (55). In our study, however, the response to DEP alone did not present clear hallmarks of enhanced T2 skewing, suggesting that the interferon associated pathways amplified by priming are independent of any interferon signalling mediated dampening response to an existing T2 dominant inflammatory milieu.

While identified in non-T2 asthma, the role of IFN pathway activation and ISG expression in the lung is often context dependent, with sometimes opposing associations with disease manifestation. This likely reflects the heterogeneity of upstream triggers from inhaled material and in the downstream balance between antiviral control and inflammatory immunopathology (56). Intriguingly in T2 dominant asthma, a role for elements of this signalling network in allergic airway disease (AAD) has also been identified. Knock out of Irf3, a transcriptional regulator of ISG expression, in mice prevented the development of HDM induced AAD through effects on dendritic cell maturation and migration and subsequent Th2 sensitization (57, 58). Although many ISG modules can restrict viral replication, enrichment of these genes alone does not establish antiviral function in T2 or non T2 asthmatic disease. Chemokines and broadly acting inflammatory genes that are also regulated by IRF signalling, including Ccl3, Ccl5 and Cxcl10 (59, 60) may instead contribute to this signature through amplification of tissue inflammation and immune cell trafficking.

An additional implication of airway states that are primed for higher IFN mediated signalling and inflammation, is how it might interact with respiratory infection, a major trigger of asthma exacerbations (61). In principle, interferon induction could improve early antiviral control. However, a primed lung in which interferon-associated inflammatory chemokines and NF-κB/Il-1/Tnf programs are simultaneously amplified could also generate exaggerated immunopathology when a viral trigger occurs. The non T2 asthma interferon literature emphasizes that interferon biology in asthma can be heterogeneous and temporally complex (56), making it particularly important to assess whether priming produces protective antiviral effects, harmful hyperinflammation, or a biphasic phenotype depending on timing and trigger order.

Our results should also be interpreted in the context of HDM as a complex biological exposure. Recent single-cell work in chronic HDM exposure models has shown interferon pathway gene expression not only in neutrophils but also in lymphoid populations and has linked major response components, including activated neutrophil states expressing Ccl3, Tnf, and Il1a, to endotoxin levels within HDM preparations (62). This supports an interpretation that at least part of the acute neutrophil response to HDM in our model may reflect responsiveness to endotoxin within HDM extracts. DEP priming could therefore amplify HDM responses by increasing the pool of responsive myeloid cells available in the airway lumen and by lowering activation thresholds within those recruited populations. Consistent with this mechanism, our *ex vivo* stimulation experiments indicated that HDM did not robustly induce several interferon-associated targets in unprimed BAL derived cells, whereas multiple interferon and inflammatory targets increased strongly in DEP-primed luminal cells, supporting a priming effect on cellular composition and responsiveness.

A major strength of this work is the separation and analysis of lung tissue, and the airway luminal cell enriched compartments, enabling inference about where pollutant conditioned programs are most prominent and which cell populations likely execute them. This dimension matters because the airway epithelium and luminal immune niche form an immunologically specialized interface that senses inhaled stimuli and gates leukocyte recruitment, positioning, and repair responses (25, 26). It also addresses a limitation of many prior DEP allergen studies in which outcomes are described in terms of airway hyperresponsiveness, remodelling, or bulk inflammatory cell counts, leaving the acute response mechanisms to inhaled exposures and changes in specific leukocyte programs less resolved (63, 64).

In our sequential exposure setting, the luminal airway compartment displayed a substantially larger magnitude of transcriptional change than whole lung tissue, consistent with the airway lumen acting as a high gain inflammatory niche that integrates recruitment and rapid responsiveness to soluble mediators at the site of exposure. At the same time, tissue contributes strongly to chemokine signals that shape luminal recruitment (29), consistent with a logic in which tissue derived mediators establish gradients and luminal infiltrates then amplify inflammation locally. While we demonstrate increases in Cxcl2 gene expression across both TISS and LAC compartments, other neutrophil chemokines Cxcl1 and Cxcl5 were much more prominent for expression in the TISS enriched compartment, consistent with a mechanism of differential recruitment signals to manage localisation of neutrophils across compartments.

In our sequential exposure model, elevated Cxcl1 and Cxcl2 protein detected in BAL after primed HDM challenge provide a direct link between transcriptomic signatures and neutrophil recruitment, through Cxcr2 mediated chemotaxis (36, 65). This mechanism is supported by previous literature. In a mouse co-exposure model, DEP exacerbated HDM-induced airway inflammation, increased BAL neutrophils and MPO, and blocking Cxcl1/Cxcl2 reduced these effects (34). DEP exposure has also been linked to macrophage driven Cxcl1 pathways as a driver of neutrophilic pulmonary inflammation in mice (66). The key advance in our current work is that a sequential priming design with a defined interval, supports a model in which DEP exposure establishes a mucosal chemokine landscape and myeloid activation state that biases how a later HDM challenge is handled, independent of direct particle allergen interaction and independent of an established sensitization microenvironment. This neutrophil associated interpretation also helps reconcile divergent literature on DEP + allergen interactions. Some studies emphasize DEP enhancement of eosinophilia and remodelling under repeated allergen challenge, consistent with a more classical T2 dominant framing (64). Other reports and mechanistic DEP models identify neutrophilic inflammation and CXC chemokine induction as dominant outcomes, particularly in settings where particulate toxicant signals, epithelial stress, and microbial associated cues converge on myeloid recruitment programs (34, 66). Our sequential design, defined timing, and compartment resolved readouts provide a mechanistic basis for how DEP can bias acute allergen responses toward neutrophil enriched programs in at least some exposure scenarios.

An additional mechanistic layer in our dataset is the co-amplification of IL-1/TNF/NF-κB signatures alongside interferon programs in neutrophil populations. Particulate exposure can induce oxidative stress and sterile inflammatory signalling that converges on NF-κB activation and IL-1 amplification (63). In DEP models, IL-1 receptor signalling has been implicated as a driver of pulmonary inflammation even when canonical NLRP3/caspase-1 processing is not the dominant upstream route (67). Other studies also link DEPs and particulate matter to inflammasome related biology in airway inflammatory contexts (68, 69). This convergence offers a coherent mechanistic proposal, in which particle induced sterile inflammation seeds IL-1/TNF mediated chemokine programs that synergize with HDM-triggered innate sensing to accelerate neutrophil recruitment and activation. Our data should also be contextualised with emerging literature describing pollutant induced neutrophil differentiation states. A recent report described SiglecF^+^ Ly6G^+^ neutrophils induced in mouse lung after DEP exposure and proposed these cells as contributors to T2/T3 inflammation and asthma exacerbation via leukotriene and NET associated pathways (70). We did not identify this phenotype in our BAL focused Scseq analysis, which may reflect differences in DEP preparation, dose, compartment analysed and/or timing. Importantly, our neutrophil populations aligned more closely with conventional neutrophil transcriptional profiles in that study, suggesting that pollutant induced neutrophil heterogeneity may be highly context and niche dependent.

Although neutrophils are a prominent component of the DEP-primed acute HDM response, our analyses also indicate that DEP conditioning reshapes macrophage and lymphoid populations. Single cell profiling showed shifts in macrophage subset composition during priming and demonstrated macrophage contribution to amplified chemokine production, consistent with broader effects in which inhaled particles may also trigger macrophage mediated effects that amplify inflammatory recruitment programs (29). These observations support a broader multicellular model in which neutrophils act alongside macrophage, NK-cell and CD8^+^ T-cell programmes rather than as a single organising centre. Indeed, it also likely there is an important contribution from the TISS compartment in governing enhanced HDM effects, potentially through altered epithelial primed states, although this hypothesis would need experimental confirmation.

Beyond classical myeloid recruitment, primed HDM exposure recruited NK- and T-cell signals and macrophage subsets consistent with alternatively activated macrophages (AAM). AAM biology is strongly implicated in airway disease pathogenesis and remodelling-related processes (71), and NK cells can modulate asthma biology in context dependent ways that include effects on sensitization and exacerbation-relevant inflammation (72). From a T2 inflammatory or allergic response perspective, it is notable that AAM like populations appeared after a single HDM dose in our priming design in the absence of strong systemic IgE changes, suggesting that early AAM programs can be initiated by innate/allergen associated cues, perhaps independent of, or upstream of classical antibody-mediated sensitization. Whether these early AAM signatures progress to stable remodelling relevant states under repeated allergen exposure remains to be determined, but their selective enhancement under primed conditions suggests that pollutant exposures can broaden the effector cell repertoire engaged even by an otherwise acute allergen trigger. Our single cell sequencing analyses also allowed us to infer chemokine signalling networks responsible for recruitment of these macrophage and lymphoid cells within the airway lumen. Ccl3 emerged as a strongly amplified chemokine in primed conditions, particularly in neutrophils, and Ccr5 linked interactions were inferred as candidate recruitment signals for AAM, T cells, and NK cells, also observed to be enhanced by DEP on HDM exposure. Importantly, Ccl3 is a potent endogenous Ccr5 ligand in some functional contexts (73), suggesting that increased Ccl3 availability could act as a strong recruitment and amplification signal for Ccr5 expressing responder populations. Ccxr3 is a well-established trafficking receptor for activated T cells and can contribute to lymphocyte recruitment in interferon-rich environments (74). Increased expression of its ligands, Cxcl9/10/11 in our data would also suggest a possible role for this chemokine network in the enhanced effects of DEP priming to recruit lymphoid and NK populations. The lack of interaction identified for this axis in the Scseq Cell chat analysis of LAC populations may indicate a cross compartment (TISS – LAC) interaction and is consistent with tissue preferential or compartment common enhanced expression of Cxcr3 chemokines.

Our data also suggest that monocyte recruitment and monocyte-derived macrophage/dendritic-cell pathways may also contribute to priming. Bulk cell type interpolation indicated increased recruited macrophage signatures (including subsets overlapping with interstitial/AAM like marker programs), and chemokine protein data implicated Ccl2 as a candidate recruitment signal for Ccr2^+^ monocytes. This is consistent with Ccr2 dependent recruitment of monocyte derived antigen presenting cells being critical in allergic airway inflammation and in DEP-associated adjuvant biology (75, 76). Within this broader framework, DEP priming may not only expand luminal neutrophils but also favour parallel monocyte recruitment/differentiation pathways that condition tissue and lumen for amplified responsiveness upon subsequent allergen stimulation.

Our compartment- and cell-resolved analyses also emphasize that priming effects can be missed by broad cytological categories. Even when total macrophage or lymphocyte counts are not dramatically altered by priming, bulk cell-type interpolation and single-cell profiling reveal redistribution within these broad classes (E.g. CD8/NK associated signals increasing under primed HDM conditions while B-cell signals decrease). Such shifts are not captured by conventional differential counts yet may foreshadow meaningful changes in antigen handling, cytotoxic/inflammatory tone, and downstream disease trajectories.

Several limitations should be considered when interpreting these findings. Our models focus on early inflammatory programs; downstream consequences for airway remodelling, hyperresponsiveness, and chronic endotype development remain to be determined. Intranasal delivery and the doses used are experimental approximations of inhalation exposure and may not map directly to real-world deposited doses. We acknowledge use of intranasal instillation as a method of exposure may result in deposition distribution and pulmonary responses that may differ from inhalation as a method of administration. We suggest these effects may be minimal, due to the specificity of response to the different materials and no evidence of substantial inflammatory response in CTRL exposures. Bulk transcriptomic readouts reflect mixed populations, and interpolation approaches, while informative cannot substitute for direct quantification of all recruited and tissue adherent populations. In addition, DEP can alter epithelial barrier properties and allergen penetration, which could contribute to priming effects alongside leukocyte mediated mechanisms and pollutant induced epithelial junction disruption as has been demonstrated in related allergic disease contexts (20). Only female BALB/c mice were examined in our study, which standardised the experimental cohort but limits our understanding of sex dependent effects. This is important as sex hormones can modify HDM induced eosinophilic and neutrophilic inflammation, IgE production and airway hyperresponsiveness, so future work should include both sexes (77). Matched fixed lung tissue was not available for histopathology because lungs were lavaged and snap frozen for molecular analysis; future studies should incorporate histopathology, airway physiology and chronic remodelling endpoints. Finally, CellChat and pathway analyses nominate, but do not prove, causal signalling. Resource constraints precluded substantial intervention studies in the present work; future experiments should test Cxcl1/Cxcl2–Cxcr2 and IL-1/TNF/NF-κB pathways using neutralising or pharmacological approaches.

## Conclusions

Collectively, our datasets support three linked conclusions. First, DEP priming is not simply additive: it qualitatively modifies the subsequent HDM response by amplifying NF-κB regulated inflammatory programs and enhancing interferon associated modules to emerge during HDM challenge. Second, these effects are strongly compartmentalized, with the airway lumen behaving as a high-gain inflammatory niche in which recruitment and per cell activation together drive large transcriptional shifts. Third, cell resolved analyses nominate Cxcr2 dependent neutrophil recruitment and neutrophil associated chemokine relays as prominent components of a broader pollutant conditioned response that also includes macrophage, NK-cell and CD8^+^ T-cell programmes.

In the broader context, a hypothesis generating translational implication of this work is that it provides mechanistic plausibility for sequential ordering of adverse inhalation exposures having an enhanced impact on airway inflammation. In real world respiratory contexts, individuals may encounter polluted traffic microenvironments followed by indoor allergen exposure (or vice versa), and sequential priming could plausibly help explain fluctuations in disease control and trigger susceptibility as well as a better understanding of how these different toxic exposures interact to worsen disease. A further translational contribution of this manuscript is that it informs what we may consider “appropriate complexity” should look like in next generation inhalation hazard testing platforms. Mechanism first frameworks such as adverse outcome pathways emphasize anchoring testing strategies on causal key events and measurable mechanistic endpoints (78). Air liquid interface models have been suggested to improve physiological relevance for inhalation toxicology and enable more realistic aerosol exposures (37, 38). However, our findings suggest that epithelial only assays may miss priming relevant biology tied to recruited myeloid cells and compartmental signalling. Advanced airway on chip systems can model neutrophil recruitment/transmigration and multicellular immune participation, offering tractable routes to incorporate the circuits emphasized here (79–81). Integrating sequential exposure designs into these platforms, explicitly separating pollutant priming from allergen challenge by biologically meaningful intervals, could enable a new class of assays to rank pollutant materials by their priming potential as well as by their standalone inflammatory potency, consistent with broader integrated testing strategies for hazard decision-making (82). These platform implications remain speculative however and as the current study did not test human prediction, material ranking or cross platform performance, prospective validation would be required before the proposed mechanisms can guide hazard decisions.

## Data Availability

The datasets used and/or analyzed during the current study are available from the corresponding author on reasonable request. The Sequencing data are deposited here: ArrayExpress, accession number E-MTAB-17021.
